# A neurocognitive model of early onset persistent and desistant antisocial behavior in early adulthood

**DOI:** 10.3389/fnhum.2023.1100277

**Published:** 2023-07-18

**Authors:** Ilse H. van de Groep, Marieke G. N. Bos, Arne Popma, Eveline A. Crone, Lucres M. C. Jansen

**Affiliations:** ^1^Erasmus School of Social and Behavioral Sciences, Erasmus University Rotterdam, Rotterdam, Netherlands; ^2^Leiden Institute for Brain and Cognition, Leiden University, Leiden, Netherlands; ^3^Department of Child and Adolescent Psychiatry and Psychosocial Care, Amsterdam University Medical Center, Vrije Universiteit Amsterdam, Amsterdam, Netherlands; ^4^Department of Developmental and Educational Psychology, Institute of Psychology, Leiden University, Leiden, Netherlands; ^5^Amsterdam Public Health, Mental Health, Amsterdam, Netherlands

**Keywords:** antisocial behavior, development, fMRI, early adulthood, self, goal-directed behavior

## Abstract

It remains unclear which functional and neurobiological mechanisms are associated with persistent and desistant antisocial behavior in early adulthood. We reviewed the empirical literature and propose a neurocognitive social information processing model for early onset persistent and desistant antisocial behavior in early adulthood, focusing on how young adults evaluate, act upon, monitor, and learn about their goals and self traits. Based on the reviewed literature, we propose that persistent antisocial behavior is characterized by domain-general impairments in self-relevant and goal-related information processing, regulation, and learning, which is accompanied by altered activity in fronto-limbic brain areas. We propose that desistant antisocial development is associated with more effortful information processing, regulation and learning, that possibly balances self-relevant goals and specific situational characteristics. The proposed framework advances insights by considering individual differences such as psychopathic personality traits, and specific emotional characteristics (e.g., valence of social cues), to further illuminate functional and neural mechanisms underlying heterogenous developmental pathways. Finally, we address important open questions and offer suggestions for future research to improve scientific knowledge on general and context-specific expression and development of antisocial behavior in early adulthood.

## 1. Introduction

Antisocial behavior, like aggression or non-compliance, violates the rights and wellbeing of others (Frick et al., 2018; see Box 1), and is costly for victims, perpetrators, and society at large (Romeo et al., 2006; Moffitt, 2018). A wealth of studies has shown that antisocial behavior peaks in adolescence (ages 10–18), and subsequently drops off during young adulthood (ages 18–26), a pattern known as the age-crime curve (Jennings and Reingle, 2012). Accordingly, young adulthood has long been recognized as a turning point for antisocial behavior, including aggression (Moffitt, 2018; Nguyen and Loughran, 2018). For most antisocial youth, early adulthood is a period where they desist from antisocial and aggressive behavior (Moffitt, 1993, 2018; Bersani and Doherty, 2018) and find their place in our society, as a result of both psychosocial and neurobiological maturation (Cauffman and Steinberg, 2000), which is associated with rising wellbeing (Arnett, 2011). However, a small subgroup persist in their antisocial behavior and show life-course persistent antisocial behavior (Moffitt, 1993, 2018), resulting in dysfunctioning in our society, and a wide range of problems later in life, including poor mental and physical health, substance abuse and involvement in crime (Shaw and Gross, 2008; Brazil et al., 2018).

**BOX 1 Characterizing aggression in social contexts.**

It is important to characterize what aggressive behavior is, while considering characteristics of the social situation or contexts, and their interactions. Ultimately, this characterization clarifies whether potential social-cognitive, behavioral, and neural deficits related to the development of aggression are general or context- and valence specific, which is important to understand the exact mechanisms underlying aggressive behavior and identify possible avenues for intervention efforts.

Aggression in social situations can have different functions

Aggression is defined as behaviors that harm others (Anderson and Bushman, 2002). Historically, two different motives for aggression have been identified: reactive and proactive aggression (Dodge and Coie, 1987). Reactive aggression occurs in response to perceived threats, provocation or frustration (e.g., social rejection), while proactive aggression is deliberate and instrumental (i.e., focused on goal-attainment) (Dodge and Coie, 1987; Card and Little, 2006; Bertsch et al., 2020). While reactive and proactive aggression have been associated with different etiologies, social-cognitive and neurobiological processes (Zhu et al., 2019, 2022), they are highly correlated and often co-occur within the same individuals (at the same time) (Card and Little, 2006). Note that particularly in (early) adulthood, reactive aggression can also be influenced by self-relevant goals [e.g., maintaining a positive and coherent self-image, reducing negative arousal or emotions (Reidy et al., 2011)].

Aggression in social contexts can have different forms

Many studies of aggression (including the ones reviewed here) have mainly focused on physical aggression [i.e., inflicting physical harm– which has been more prevalent and normative in males (Nelson et al., 2008)]. However, throughout the course of development, adolescents and early adults (especially females) increasingly use more subtle forms of aggression (often called social or relational aggression), like non-verbal aggression, or spreading rumors (Vitaro et al., 2006; Nelson et al., 2008).

Aggression in social contexts have a level of directness

Aggression can vary in the level of directness (also known as overt vs. covert aggression). More specifically, a higher level of direct (overt) aggression means that the target is present and identifiable. Females often use less direct forms of aggression than males (Nelson et al., 2008), such as gossip and rumors.

Aggression in social contexts can involve different (amounts of) people

Aggression can be shown by and toward different targets, with varying levels of familiarity (e.g., peers, romantic partners, unknown others). Characteristics of the target are often important (e.g., gender seems associated with different types of social norms that need to be adhered to, Nelson et al., 2008). Likewise, the amount of people simultaneously involved in a provocative act influences behavioral and neural responses (e.g., social rejection (involving one person at the same time) is experienced differently than exclusion (involving more than one person at the same time) (Rappaport and Barch, 2020).

One important factor that has been proposed to differentiate between persistent and desistant antisocial developmental trajectories concerns differential patterns of brain development (Moffitt, 1993, 2018). In line with this idea, recent studies have shown that life-course persistent antisocial behavior–but not desistant trajectories–was characterized by differential cortical and subcortical brain structure (Carlisi et al., 2020, 2021). However, until recently, possible functional mechanisms that help explain how and why differences between persistent and desistant antisocial developmental trajectories arise remained largely elusive, especially in young adulthood (van de Groep et al., 2022a,b). In the current review, we highlight and summarize recent functional neuroimaging studies on social-cognitive information processing (self- and other oriented) showing that, compared to desistant and non-antisocial behavior, persistent antisocial behavior in early adulthood is largely characterized by distinct difficulties in social cognitive functions and related disturbances in underlying brain functioning (Brazil and Buades-Rotger, 2020). We particularly emphasize developmentally salient difficulties specific to early adulthood, focusing on how young adults evaluate, act upon, monitor, and learn about their goals and self traits. Moreover, we integrate these findings with existing theoretical frameworks and recent studies on adolescent neurocognitive development to formulate a neurocognitive working model of persistent and desistant antisocial development from adolescence into early adulthood. Given that most research on the characteristics and mechanisms underlying the persistence of antisocial behavior has been conducted in males (Moffitt, 2018; Eme, 2020; but see Freitag et al., 2018), most findings discussed in this review focus on male antisocial behavior.

First, in Section “1.2. The neurodevelopment of childhood-limited persistent and desistant trajectories of antisocial behavior in late adolescence,” we describe the current knowledge on neurodevelopmental differences between persistent and desistant antisocial trajectories in late adolescence. Subsequently, in Section “1.3. Neurodevelopmental changes in early adulthood,” we focus on the typical neurodevelopment in adolescence and early adulthood. In Section “1.4. Social information processing theory of aggressive and antisocial behavior,” we introduce social information processing theory and in Section “1.5. Toward a neuropsychological working model of early adulthood antisocial behavior” we discuss why an integrated neuroscience model of early adulthood antisocial development is warranted. Next, in Section “2. Current evidence of behavioral and neurobiological social information processes involved in the development of aggressive and antisocial behavior in early adulthood,” we integrate recent empirical findings to highlight behavioral and neurobiological social information processes that may underlie differences in the development and maintenance of, and desistance from aggressive behavior. To frame our discussion, we combine insights from the developmental taxonomy of antisocial behavior (Moffitt, 1993, 2018), social information processing theory (SIP, Dodge and Crick, 1990; Crick and Dodge, 1994) and the psychosocial maturation model (Cauffman and Steinberg, 2000), with recent neuroimaging findings. Finally, in Section “3. Working model and future directions” we describe the working model of early adulthood antisocial behavior and highlight important areas for future research. Note that this review was specifically aimed at a novel direction of research on social-cognitive processes in early adulthood and therefore was not intended to include all research on antisocial individuals to date.

### 1.2. The neurodevelopment of childhood-limited persistent and desistant trajectories of antisocial behavior in late adolescence

One of the most influential and well-cited theories on persistent and desistant antisocial development is the taxonomy proposed by Moffitt (1993, 2018), which outlined two distinct developmental trajectories of antisocial behavior. The first, known as life-course persistent antisocial behavior, emerges early in life, and is characterized by early neurodevelopmental problems, which are repeatedly amplified and reinforced by a high-risk social environment throughout development (Moffitt, 2018). The second trajectory, known as adolescence-limited antisocial behavior, emerges in adolescence, and is thought to result from developmentally normative desire to feel more mature (Monahan et al., 2013). In addition to these antisocial groups, there are also individuals who abstain from antisocial behavior throughout development (Frick and Viding, 2009; Monahan et al., 2013; Moffitt, 2018). Longitudinal research generally supports the taxonomy, but additional developmental trajectories have also been identified (Piquero, 2008). One such trajectory consists of individuals with childhood-limited of antisocial behavior who show conduct problems early in life, but desist in adolescence and early adulthood (Monahan et al., 2013; Bevilacqua et al., 2018; van de Groep et al., 2022a,b). Whether antisocial behavior is present and persistent can be measured and operationalized in different ways, with two common approaches involving classification based on clinical, psychiatric diagnoses and symptoms, and classification based on offending patterns based on police registrations (Bersani and Doherty, 2018; Moffitt, 2018). In the current review, we focus on studies that used a clinical classification of antisocial development based on diagnostic interviews (see also Box 2), to differentiate between (1) young adults who persist from childhood to early adulthood, (2) young adults who showed antisocial behavior in childhood but no longer in early adulthood (van de Groep et al., 2022a).

**BOX 2 Understanding the development of antisocial behavior and psychopathic traits.**

Severe antisocial behavior has been linked to various clinical diagnoses and symptoms in children, adolescents and adults. The diagnoses and their criteria differ depending on the age of the individual, in line with the observation that social norms, and hence potential violations of these norms, differ between developmental groups (Pauli and Lockwood, 2022). In childhood and adolescence, youth can be diagnosed with Conduct Disorder and Oppositional Defiant disorder, which are both characterized by antisocial behaviors and defiance, irritability and anger, and irresponsibility (Pauli and Lockwood, 2022). After the age of 18, adults can receive the diagnosis of Antisocial Personality Disorder (ASPD), which is likewise characterized by criminal behaviors, irritability and anger, and irresponsibility, as well as impulsivity and a lack of remorse or guilt (Pauli and Lockwood, 2022). ASPD is seen as an heterogenous disorder, which is often sub-typed based on the presence or absence of psychopathic and other personality traits (Marsden et al., 2019). Psychopathy is a personality construct characterized by difficulties in affective, interpersonal and behavioral domains (Carré et al., 2013; Nentjes et al., 2022), and is sometimes considered a particularly severe subtype of ASPD (but see Blair, 2022a,b), with emotional deficits and a lack of remorse likely being more central in psychopathy than ASPD (Blair, 2022a,b). Approximately 30% of adults with ASPD also meet the criteria for Psychopathy, while most individuals with Psychopathy meet the criteria for ASPD (Sarkar et al., 2011; Blair, 2022a,b).

Although there are many different conceptualizations and operationalizations of psychopathy in the literature, most concur on the notion that psychopathy is multidimensional in nature (Lilienfeld, 2018). Research on the development of psychopathic traits often employs a conceptualization consisting of three dimensions: Callous-Unemotional traits, Impulsive-Irresponsible traits and Grandiose-Manipulative traits (Andershed et al., 2002), which has received ample empirical support (Lee and Kim, 2020). At the same time, many studies using this conceptualization have focused predominantly on either the total, global construct of psychopathy, or only one of the dimensions of psychopathy (Callous-Unemotional traits) (Lilienfeld, 2018)–as measured with either the Youth Psychopathic Trait Inventory (YPI; Andershed et al., 2002) or the Inventory of Callous Unemotional Traits (ICU; see Cardinale and Marsh, 2020 for a meta-analysis on the reliability and validity of the ICU; see also Ansel et al., 2015 for associations and differences between these self-report measures).

However, it has become increasingly clear that the different dimensions of psychopathy are often associated with different behavioral and neurological outcomes and mechanisms and thus potentially provide information above and beyond other dimensions (Carré et al., 2013; Lilienfeld, 2018). Coincidently, in some situations, psychopathic dimensions may also interactively influence such outcomes and mechanisms (Lilienfeld, 2018) and show uniformity across dimensions (Garofalo et al., 2018). Although psychopathic traits tend to show a relatively stable pattern throughout development, recent developmental studies emphasize the potential for change in some individuals, and different expressions in changing social contexts as adolescents transition into early adulthood (Lee and Kim, 2020; Nentjes et al., 2022).

Alongside other longitudinal studies (see e.g., Poulton et al., 2015; Carlisi et al., 2020), a Dutch longitudinal childhood arrestee cohort study, called RESIST, has started to identify when and why developmental outcome differences arise between groups with an early onset of antisocial behavior in adolescence and early adulthood (van de Groep et al., 2022a,b), with a special focus on psychological and neurobiological functional mechanisms. To study functional mechanisms that underlie differences in mental and behavioral functioning between developmental groups in adolescence, Cohn and colleagues examined a subsample of the childhood arrestee cohort of the RESIST study at age 17 and used functional magnetic resonance imaging (fMRI). As expected, this approach revealed several mechanisms contributing to persistence, including deficient processing of feedback cues. First, using an adapted version of the monetary incentive delay task, Cohn et al. (2015) showed that persistence, but not desistance of antisocial behavior was associated with hypoactivity in the ventral striatum during reward processing, and with amygdala hyperreactivity during loss processing (see Box 3 for an overview of the role of the amygdala in early adulthood antisocial behavior). These alterations raise the question whether such aberrant incentive processing underlie difficulties in (reinforcement) learning and decision making. Second, Cohn et al. (2013) and Cohn et al. (2016a,b) also revealed that some behavioral and neural patterns were not specific to persistent antisocial behavior, but instead underlie both persistent and desistant developmental groups. For instance, both persistent and desistant antisocial behavior were characterized by neural hyperactivity during fear acquisition and extinction, compared to healthy controls. Together, these findings suggest that both persistent and desistant antisocial behavior may be associated with altered salience processing of negative (feedback) cues (Cohn et al., 2015) – although this pattern may be more prevalent (across different contexts) for persistent antisocial behavior.

**BOX 3 The role of the amygdala in early adulthood antisocial behavior.**

Researchers have long suggested that antisocial behaviors across the lifespan may result from a failure to appropriately interpret and use social cues from others (Blair, 2005; Marsh et al., 2008). For instance, antisocial behavior may result from problems with processing of distress and threat related cues, empathy and mentalizing (Marsh et al., 2008; Blair, 2013). Many theories have suggested that these difficulties result from reduced activity in the amygdala (see e.g., Blair, 2013). In line with these theories, a recent meta-analysis revealed that youth (ages 10–21) with conduct problems indeed show reduced right amygdala activation in response to negatively valenced images and fearful expressions (Berluti et al., 2023). However, several other meta-analyses found no evidence that youths and adults (ages 10–44) showed amygdala hypoactivity compared to typically developing controls (Alegria et al., 2016; Dugré et al., 2020).

To reconcile these conflicting findings, some researchers have suggested that amygdala hypoactivity is particularly likely to occur if high levels of psychopathic traits – and particularly Callous-Unemotional traits – are present alongside conduct problems or antisocial personality disorder (Viding and Larsson, 2007; Poeppl et al., 2019; Dugré et al., 2020). However, this effect is not consistently found across studies and meta-analyses (Deming et al., 2022; Berluti et al., 2023), as most studies on psychopathic traits and amygdala structure or function report null-effects instead (Deming et al., 2022). The inconsistency across studies, and proliferation of null-findings might be due to methodological characteristics like limited power, sample population, imprecise labeling of peak coordinates, and suppression effects of different psychopathic trait sub-dimensions (Deming et al., 2022). Alternatively, findings of reduced amygdala responsiveness may be specific to adolescence (Blair, 2022a,b), signaling the need to test whether and how developmental effects may unfold over time, and especially during early adulthood. Yet another possibility is that amygdala activity in both typically developing and antisocial young adults is context-dependent (Dotterer et al., 2017; Gothard, 2020; Deming et al., 2022), and may be involved in fine-tuning and flexibility of social functions in different and changing social situations. Although this finding fits with lesion studies (Gothard, 2020), studies in human youth and adults so far have not found evidence for this hypothesis (Deming et al., 2022). Taken together, additional research is necessary to elucidate the role of the amygdala in early adulthood antisocial behavior, including the role of developmental phase and context factors.

Similar to an early onset of antisocial behavior, psychopathic personality traits have also been associated with a more severe and persistent development of antisocial behavior (Stickle et al., 2009; Frick et al., 2014; Cohn et al., 2015, 2016a; see Box 2). Interestingly, separate dimensions of psychopathic traits, such as Callous-Unemotional, Grandiose-Manipulative and Impulsive-Irresponsible traits (Andershed et al., 2002) have been shown to influence neurocognitive functioning in persistent and desistant antisocial development. For instance, in prior functional neuroimaging work in adolescents by Cohn et al. (2013, 2015, 2016a), neural hyperactivity during fear learning and extinction was positively associated with Impulsive-Irresponsible traits in persistent and desistant antisocial groups, while Callous-Unemotional traits were negatively associated with neural responses during reward processing and fear conditioning. In line with these findings, structural imaging studies also revealed distinct structural patterns in similar limbic brain areas (Insula, Amygdala) for adolescents characterized by Callous-Unemotional traits (Cohn et al., 2016b). Moreover, Callous-Unemotional traits and Grandiose manipulative traits showed unique patterns of structural connectivity (Pape et al., 2015). Notably, high levels of psychopathic traits do not only affect brain structure and functioning, but also affect associated (mal)adaptive behavior, where higher levels may be beneficial in some types of situations (e.g., fast-life strategy; quickly changing or hostile social contexts, Doerfler et al., 2021), and more negative in others (e.g., situations that require multi-tasking or processing of multiple information streams) (Baskin-Sommers and Newman, 2013; Doerfler et al., 2021). Combined with the empirical observation that early adulthood is a salient period for the development and influence of personality traits and personality disorders on aggression (Ostrov and Houston, 2008) - like psychopathy and Antisocial Personality Disorder (ASPD) – these findings suggest that considering psychopathic traits may offer additional clues about mechanisms underlying the persistence of antisocial behaviors into early adulthood, and help explain the observed heterogeneity in antisocial developmental trajectories (Brazil et al., 2018).

Even though important prior work focused on antisocial development in adolescence (Frick and Viding, 2009; Blair, 2013; Moffitt, 2018), relatively few studies have focused on the transition from adolescence into early adulthood, despite evidence that this developmental period is perhaps equally important to understand the (dis)continuation of antisocial behavior throughout the life course (Monahan et al., 2013; Taber-Thomas and Pérez-Edgar, 2015). This long-term developmental approach, despite being practically challenging, is one of the only ways to examine persistent versus desistant trajectories in development (Moffitt, 1993, 2018). Eventually, an improved understanding of desistance may offer informative clues on how to improve treatment and interventions for those who show antisocial behavior throughout development (Dotterer et al., 2023). Before considering potential neurocognitive differences between these trajectories, and potential impairments or adaptations that are associated with these developmental pathways, we will now turn to describing typical neurodevelopmental changes in early adulthood.

### 1.3. Neurodevelopmental changes in early adulthood

Early adulthood is a life period that is characterized by changes in social interactions, due to rapidly changing environments, social relationships, social roles, and social norms (Arnett, 2000, 2007; Sussman and Arnett, 2014). The exact age ranges for early adulthood are dependent on contextual factors such as societal norms and historical times, but general consensus is that early adulthood encompasses approximately the age ranges 18–26 (Sawyer et al., 2018). To navigate these contextual changes and ultimately effectively function as an adult in society, young adults need to develop knowledge, skills, and self-understanding to balance between environmental constraints and their own goals (Arnett, 2000, 2007). Developmentally distinctive to other developmental periods across the lifespan is that early adults focus more on themselves, individualistic goals (Nelson, 2021), and the development of various (social) identities that fit different social roles and contexts (Arnett, 2000). During early adulthood, individuals also grow more confident that they can achieve their goals (i.e., period of opportunities and possibilities, Arnett, 2000).

**FIGURE 1:**
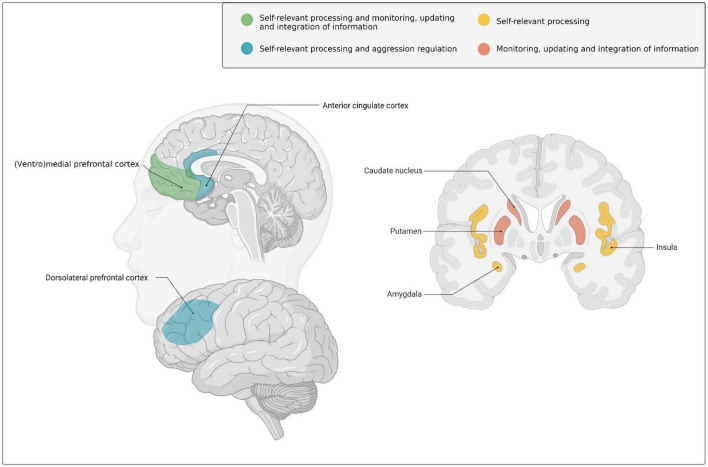
Schematic overview of the brain regions that undergo marked neurodevelopmental changes during the transition from adolescence into early adulthood and have been implicated–amongst other functions- in self-relevant and goal-related social information processing, behavioral regulation, monitoring and learning in early adulthood. Created with BioRender.com.

These social and psychological changes during early adulthood are supported by ongoing brain development (Taber-Thomas and Pérez-Edgar, 2015; Mills et al., 2016; Tamnes et al., 2017; Herting et al., 2018). More specifically, early adulthood is marked by extensive structural changes in association cortices (i.e., areas that integrate and associate information from various sensory modalities) and frontolimbic systems (Casey et al., 2005, 2019), such as the prefrontal cortex (PFC) [in particular in the ventrolateral and dorsolateral PFC, as well as the ventromedial PFC extending into the anterior cingulate cortex (ACC)], and subcortical limbic structures like the (ventral) striatum and insula (For an extensive overview of brain development in early adulthood including structural MRI and postmortem evidence, see Taber-Thomas and Pérez-Edgar, 2015; see also Figure 1 for a visual illustration of brain areas involved in development across early adulthood).

Not only in structural, but also in functional brain development early adulthood marks a period of transition. During the transition from adolescence into early adulthood, individuals show increased prefrontal functioning and enhanced connectivity between the dorsal and ventromedial PFC and subcortical structures (e.g., striatum), as well as other cortical structures (e.g., parietal cortices see Casey et al., 2008; Taber-Thomas and Pérez-Edgar, 2015; Bos et al., 2020). These neurobiological changes during early adulthood are thought to provide top-down subcortical modulation to overcome the imbalance of brain corticostriatal circuitry that often drives behavior in adolescence (Casey et al., 2008; Mills et al., 2014; Casey, 2015). As such, increased brain maturation and connectivity may facilitate several processes that may be important for the development of and desistance from antisocial behavior, including integration of multiple streams of cortical and subcortical (social) information processing, appropriate behavioral selection, behavioral regulation/self-control (e.g., balancing between approach and avoidance tendencies), including emotion regulation (Casey, 2015; Andrews et al., 2021), and future-oriented behavior [e.g., increased attention toward and opportunities to learn from negative (long-term) consequences] (Monahan et al., 2013; Casey, 2015; Flechsenhar et al., 2022). Together, these social, psychological, and neurobiological changes in early adulthood support adaptation to constantly changing environments (Casey, 2015; Andrews et al., 2021; Flechsenhar et al., 2022), while balancing these situational characteristics with self-relevant goals and motivations.

### 1.4. Social information processing theory of aggressive and antisocial behavior

Studying the role of social cognitive processes contributing to aggressive and persistent antisocial behavior is important to understand the origin and maintenance of such behavior (Choe et al., 2015), as well as to improve assessment, and ultimately prevention and intervention efforts (Klein Tuente et al., 2019). The Social information processing model (SIP, Dodge and Crick, 1990; Crick and Dodge, 1994) provides a theoretical framework to understand aggressive antisocial behavior in social contexts in early adulthood. Note that although antisocial behavior in social contexts can be expressed in various non-aggressive manners (e.g., rule breaking, theft, vandalism), the majority of neurocognitive theories and studies have focused on aggression specifically (Tremblay, 2010; Dugré et al., 2020; see Box 1). In line with this observation, most of all studies included in this review will focus on aggression as a specific manifestation of antisocial behavior.

According to SIP, how aggressively someone responds to social cues depends on both the social cues themselves, as well as on how they are interpreted and processed. Over the past few decades, the study of social information processing has greatly informed the understanding of both aggression in specific social contexts, and the development and maintenance of persistent antisocial behavior (Stickle et al., 2009), especially in childhood and adolescence (Bowen et al., 2016; Klein Tuente et al., 2019). According to the SIP model, social information is interpreted and responded to in six steps (Dodge and Crick, 1990; Crick and Dodge, 1994): In the first step, individuals *attend to and encode information* from the current social situation, using internal (physiological and emotional states) and external cues (environmental stimuli). Second, individuals *give meaning to the information*, using contextual cues and organized knowledge from memory. During this step, individuals interpret the intent of others [e.g., hostile attribution bias (Klein Tuente et al., 2019; Smeijers et al., 2019)], and consider what the situation might mean for self and others (Galán et al., 2022). In the third step, individuals *set a specific goal* for the current situation. Fourth, individuals *identify potential responses* for the current situation (either accessed from long-term memory based on previous stimulus-response associations, or newly generated). During the fifth step, individuals *evaluate whether the response* chosen in step 4 *is indeed the best to select and implement* (e.g., by considering the anticipated consequences of behavior). Finally, *the selected response is performed and monitored*. Together, the first three steps concern social cognitions about input, while the final three steps are social cognitions about output (Bowen et al., 2016). Note that the order of SIP is not sequential, but cyclic with multiple feedback loops and often simultaneous processed (Galán et al., 2022).

### 1.5. Toward a neuropsychological working model of early adulthood antisocial behavior

In the current review, we will use the SIP model as starting point to investigate developmentally salient features of early adulthood regarding social information processing, by examining the interplay between SIP steps. Although most research on SIP has been done in children and adolescents (Bowen et al., 2016; Klein Tuente et al., 2019), this model may provide an important framework to understand the social-cognitive processes and the neural basis of social information processing and reactive aggression in early adulthood (Vitaro et al., 2006). Different parts of the model fit well with specific developmentally salient characteristics and changes of early adulthood and may provide important starting points for research into persistent and desistant antisocial trajectories.

Prior studies examining neural responses to social stimuli have mainly focused on separate – and usually the first – information processing steps (e.g., encoding and interpretation; Dodge et al., 2006). For example, many studies have examined neural responses to emotional faces and social threats in the amygdala (Blair, 2022a), a deep subcortical emotion processing region (Adolphs, 2010; Bickart et al., 2014; Bertsch et al., 2020). These studies showed that the amygdala is involved in recognizing emotions from faces, and more generally contributes to the processing of emotional and socially relevant, salient information (see e.g., Sergerie et al., 2008; Adolphs, 2010; for reviews and meta-analyses). Notably, apart from emotional responsiveness, most research to date has focused on other forms of empathic responsiveness (Blair, 2005, 2022a; Jones et al., 2010), including the perception and attribution of intent and beliefs to others (e.g., Bertone et al., 2017; Blair, 2022a), perspective taking (e.g., Lui et al., 2016), and responses to other people’s pain (e.g., Marsh, 2013). As such, research has been largely ignoring the hypothesized role for internal, self-related processing in antisocial development (Dodge and Crick, 1990; Crick and Dodge, 1994; Huesmann, 1998; but see Blankenstein et al., 2021), at least with regard to self-evaluation (i.e., SIP step 2, van de Groep et al., 2022b).

Moreover, only recently have researchers examined the interaction between multiple social information processing steps, including between the first steps (encoding, interpretation, goal selection) and the second steps (identification, selection, monitoring) (Dodge et al., 2022; van de Groep et al., 2022a,b). In line with this notion, researchers have called for an extension of the SIP model to more closely integrate emotion and cognition across all SIP steps (Lemerise and Arsenio, 2000; Smeijers et al., 2020). Emotional processes (e.g., emotional experiences, emotional understanding, emotion recognition, and emotion regulation, Smeijers et al., 2020) are thought to reduce information processing demands and support goal-directed behavior (Lemerise and Arsenio, 2000; Smeijers et al., 2020). Indeed, impaired emotional processes do not only affect the first two–but rather all SIP steps, probably also interactively, and have been shown to contribute to (the development of) aggressive behavior (Lemerise and Arsenio, 2000; Smeijers et al., 2020; Blair, 2022a,b).

Therefore, in the current review, we will focus on how young adults evaluate, act upon, monitor and learn about themselves (i.e., their self traits, or self-concept) and self- and other-related goals, and the neural underpinnings of these processes and behaviors. For social cognitive input processes, we differentiate between internal and external processes. With internal processes, we refer to evaluating and monitoring traits and actions of self, also referred to as self-appraisals. By external processes we refer to performing and monitoring actions in response to or affecting others (e.g., social evaluations). Regarding social information output, we will specifically consider how people learn action-outcome associations for self and others and how they (fail to) regulate aggressive responses to social feedback. For each of these processes, we will focus on positively, negatively and/or intermediately valenced information, in general developmental patterns in young adults with and without a history of antisocial behavior (either persistent or desistant, Moffitt, 1993, 2018; van de Groep et al., 2022a,b), and additionally discuss potential associations with psychopathic traits.

## 2. Current evidence of behavioral and neurobiological social information processes involved in the development of aggressive and antisocial behavior in early adulthood

### 2.1. Encode, interpret, and integrate self-relevant information

#### 2.1.1. Evaluating the self

In most individuals, their view of themselves (i.e., their self-concept) is generally positive and well-structured, with a more positive self-concept in some domains than others (see Crone et al., 2022 for a systematic review). A positive self-concept has been associated with increased wellbeing and self-efficacy (Rodman et al., 2017), and its development is largely shaped by previous social experiences and development of cognitive abilities (Crone et al., 2022). Positivity of the self-concept, however, temporarily dips in adolescence, and rises again into early adulthood (Rodman et al., 2017; Moses-Payne et al., 2022; van der Cruijsen et al., 2023). This positivity rise in early adulthood might be due to an increased reliance on accumulated rather than immediate social feedback from others (Will et al., 2017; Yoon et al., 2018).

Having a well-structured self-concept, that is both relatively stable and malleable at the same time, helps young adults to establish continuity and goal-attainment in a changing social world, and to adjust their behavior to the possibilities (opportunities and constraints) of the social situation (Crone et al., 2022). The self-concept has been divided into two parts: self-concept appraisal (i.e., the estimated qualities or attributes of the self), and self-concept clarity (i.e., “the extent to which knowledge and beliefs about the self are clearly and confidently defined, internally consistent, and temporally stable”) (Crone et al., 2022). Together, these findings show that adolescence and emerging adulthood are important periods in which self-concept is shaped by experiences.

In terms of SIP, self-evaluations can be separated in immediate feelings concerning traits of self (encoding interpretation, goal selection) and selection and modification or our immediate response to self-appraisals (identification, selection, monitoring). Given the complexity of self-related thoughts and their sensitivity to biases, studying self-appraisals is inherently complex. One of the key developmental tasks during adolescence and early adulthood is to develop a clear and coherent sense of the multidimensional, and increasingly complex self (Branje et al., 2021). During typical development, self-concept clarity typically rises during young adulthood (Wu et al., 2010; but see Crocetti et al., 2016a,b), with individuals becoming more confident and consistent in their self-beliefs, although considerable heterogeneity between young adults has been reported (Branje et al., 2021).

Although very few studies have focused on the neural underpinnings of self-concept clarity (van der Aar et al., 2019; Xiang et al., 2022), the first evidence suggest that the mPFC play an important role in maintaining a coherent self-image (for ventral and anterior/rostral mPFC see Elder et al., 2021; for precentral gyrus see Xiang et al., 2022). Neuroscience studies have examined self-appraisal using trait-evaluation paradigms, by asking participants whether positive and negative trait statements fit with them, in different domains (van der Cruijsen et al., 2017, 2018). Using these paradigms, previous research has repeatedly shown activity during self-appraisals in cortical midline areas (Northoff et al., 2006; Denny et al., 2012). In particular, the anterior medial prefrontal cortex (mPFC) often shows increased activity for self-related activation (Northoff et al., 2006; Denny et al., 2012). Some studies suggest that rostral mPFC activity decreases between adolescence and early adulthood in response to (neutral) self-related stimuli, a finding that has been interpreted as reflecting requirement of less processing capacity – and hence as more efficient processing, due to increased maturation (Davey et al., 2019).

More detailed understanding of self-appraisals has been acquired by examining self-appraisals in different contexts. First, developmental comparison studies have pointed to increasing domain differentiation in activity in mPFC when evaluating self traits. Indeed, mPFC activity has been shown to depend on the valence of self-related stimuli (van der Cruijsen et al., 2017; Van de Groep et al., 2021), with stronger activity for positive traits (van der Cruijsen et al., 2017), and/or traits that are more applicable to the self (D’Argembeau, 2013). Furthermore, early adults show differentiation in self-appraisal across different life domains (e.g., social, physical, academic domain (van der Cruijsen et al., 2018; Van de Groep et al., 2021). For instance, young adults generally find positive prosocial traits to be more applicable to self than positive physical appearance traits (van der Cruijsen et al., 2018; van der Aar et al., 2019; Van de Groep et al., 2021). Moreover, evaluations in diverging domains have also been associated with different neural underpinnings (see van der Cruijsen et al., 2018; Van de Groep et al., 2021). Interestingly, having a complex, multi-faceted self-concept may act as a buffer to maintain positive self-views (Rodman et al., 2017). More specifically, if negative feedback threatens one specific domain (e.g., social domain), young adults likely draw upon alternative sources of positive feelings of self-worth from other domains (e.g., physical appearance domain, Rodman et al., 2017). Hence, in typically developing young adults, self-appraisals become more increasingly multifaceted and complex, which support both goal attainment and adaptation to changing social contexts.

An important question concerns whether early adults with a history of aggressive behavior form similar neural responses to self-evaluation across domains, especially given that one’s self-concept is shaped by social experiences (Harter, 2012). Behavioral studies show that individuals with more antisocial behavior have a lower self-esteem (Donnellan et al., 2005). Likewise, self-concept clarity is negatively associated with aggression in (early) adults, both in typically developing samples and incarcerated adults (Steffgen et al., 2007; Edwards and Bond, 2012). Moreover, research on early adults with higher levels of psychopathy and prior reports of delinquency not only report weaker or more instable self-beliefs (Levey et al., 2019; Doerfler et al., 2021), but also suggest that self-concept clarity may influence the speed of desistance from antisocial behavior (Levey et al., 2019). More specifically, adolescents with a lower self-concept clarity desist from delinquency at a later age (Levey et al., 2019).

Few studies to date examined self-appraisals in relation to antisocial experiences. In a recent study, we used a trait-based self-appraisal paradigm in young adults with childhood-limited persistent antisocial behavior, and childhood-limited desistant antisocial behavior and compared them with typically developing young adults (van de Groep et al., 2022b). The study confirmed increased activity in mPFC for self-appraisals, consistent with prior studies (Denny et al., 2012; van der Cruijsen et al., 2018) and showed that the same brain regions are recruited for self-appraisals across groups with various histories of antisocial behaviors. Across the total sample, psychopathic traits (combination of Callous-Unemotional traits, Grandiose-Manipulative traits and Impulsive-Irresponsible traits) were associated with more negative and less positive self-appraisals in the prosocial domain, and not in the physical appearance domain. In terms of neural activity, Callous-Unemotional traits were associated with less amPFC activity during general self-evaluations, which might signal differences in how individuals with higher levels of Callous-Unemotional traits constrain abstract information during conscious experiences that involve thinking about the self, possibly to increase the stability of thought in line with existing cognitive schema’s about the self (Zamani et al., 2022). Taken together, these findings suggest that the super-ordinate construct of psychopathy is associated with domain-specific self-appraisals, while specific sub-dimensions (e.g., Callous-Unemotional traits) show distinct neurobiological functional alterations across domains – highlighting that considering both total levels of psychopathic traits and specific subdimensions in future research may reveal more insights into the etiology and complex pathways related to antisocial behavior.

### 2.2. Learning about the self

A second step in social information processing is our immediate response to feedback from others that may affect our self-appraisal. Often, individuals employ strategies to protect their self-image, such as retaliation or down-grading the messenger. Thus, how we evaluate ourselves is influenced by (1) evaluating social feedback that we receive from others and (2) responding to social feedback and (3) goals and motivations that we hold (e.g., view ourselves positively and maintain coherent and consistent view about ourselves (Elder et al., 2021; Crone et al., 2022).

#### 2.2.1. Evaluation of social feedback about the self

Social feedback can signal positive or negative information about oneself or one’s behavior. Receiving social feedback is important for learning, imitation, and adaptation of social behavior (Zhang et al., 2022), and the pursuit and attainment of goals (Fishbach and Finkelstein, 2012). Social feedback can take many forms that differ depending on the number of people involved and the content or type of feedback (Rappaport and Barch, 2020). Most studies on social feedback have focused on neural activation underlying social exclusion (i.e., negative social feedback by multiple individuals at the same time), which is often assessed using the Cyberball task [ Williams and Jarvis, 2006; for meta-analyses see (Cacioppo et al., 2013; Vijayakumar et al., 2017; Mwilambwe-Tshilobo and Spreng, 2021)], by contrasting this to neural activation to social inclusion. Generally, negative social feedback triggers anger and frustration, which in turn leads to reactive aggression (Dodge et al., 2003; Chester and DeWall, 2014). Studies using the Cyberball paradigm typically reveal that social exclusion evokes increased activity in cortical midline areas like the ACC, mPFC and anterior insula (see Cacioppo et al., 2013; Vijayakumar et al., 2017).

The responses in the ACC, mPFC and anterior insula have been interpreted as reflecting “social pain” (Eisenberger and Lieberman, 2004) as they respond strongly to social rejection, or “salience” as the ACC is also active when social feedback does not match prior expectations (Somerville et al., 2006). However, recent findings indicate that activity in these areas may not be specific to negative social feedback (i.e., not valence-specific), but instead reflect increased social salience of all stimuli that elicit affective responses, including positive feedback (Dalgleish et al., 2017; Perini et al., 2018; Van de Groep et al., 2021). Moreover, the Cyberball inclusion condition is often considered to be a neutral, rather than a positive and rewarding condition (Rappaport and Barch, 2020) meaning that it includes only one salient event (Perini et al., 2018). Therefore, studies have introduced experimental tasks that do not only distinguish between negative and neutral feedback, but also positive feedback (Guyer et al., 2008; Silk et al., 2012; Kujawa et al., 2014). The Social Network Aggression task (SNAT, Achterberg et al., 2016), is a task in which participants receive positive, negative, and neutral feedback from their peers, and subsequently get the opportunity to show or regulate aggressive behavior toward the sources of social feedback by sending a (not so) loud noise blast. Several studies in young adults employing the SNAT show that both positive and negative feedback elicit activity in the ACC (Achterberg et al., 2016), AI (Achterberg et al., 2016; Van de Groep et al., 2021) and (v)mPFC (Achterberg et al., 2016; Van de Groep et al., 2021), compared to neutral feedback. Taken together, receiving social feedback from others results in activity in a network of “salience” brain regions, including the ACC and insula, that may signal importance of the events.

To address the question whether youth with various histories of antisocial behavior interpret feedback from others differently than early adults without a history of antisocial behavior, neuroscience studies may provide a direct marker of salience. From a SIP perspective, young adults with prior antisocial experiences may interpret neutral (intermediate or mixed feedback, signaling an ambiguous situation) as more hostile and indicative of rejection (Crick and Dodge, 1994; Dodge et al., 2003; Brennan et al., 2018), and hence more salient and self-relevant, which is reflected in neural hypersensitivity to cues signaling potential social rejection (Baskin-Sommers and Newman, 2013; Blair, 2013; van de Groep et al., 2022a). In a recent study, we studied social feedback processing in young adults with childhood-limited persistent antisocial behavior, childhood-limited desistant antisocial behavior and typically developing young adults, using the SNAT (van de Groep et al., 2022a). Early adults with a history of prior antisocial behavior (persisters and desisters) showed increased AI activity during feedback processing, regardless of feedback type (van de Groep et al., 2022a), compared to the healthy controls. Possibly, this finding reflects difficulties in the ability to differentiate between social feedback cues (Kawamoto et al., 2015a,b). An additional finding was that increased activity in the dlPFC during general feedback processing was specific to the desisting group (van de Groep et al., 2022a). This increased dlPFC activity likely reflects attentional processes in response to changing task demands (context-dependent changes in feedback presentation between trials; Niendam et al., 2012; Bertsch et al., 2020), which support cognitive and emotional regulation of subsequent behavior (van de Groep et al., 2022a)–and thus helps young adults with a desisting developmental trajectory to refrain from aggressive behaviors, if such behavior is appropriate in the specific situation.

It is important to note that the effects on insula and DLPFC activity were not specific to the valence of the stimuli, but were general (i.e., observed independent of whether the signaled feedback was positive, neural or negative). Interestingly, a recent study suggests that ambiguous social contexts may be better suited to reveal individual differences in behavior and neural activity related to antisocial behavior than unambiguous ones (Brennan et al., 2018). More specifically, a recent EEG study in adolescents and young adults demonstrated that higher levels of psychopathic traits were associated with more elaborate social information processing in ambiguous social contexts (Brennan et al., 2018), but not unambiguous social contexts (e.g., the exclusion condition in the cyberball task). Hence, to further elucidate heterogeneity in aggressive behavior, studying behavioral and neural responses related to different ambiguous aspects of social situations (e.g., indirect aggression) is an important avenue for future research.

Taken together, processing feedback of others on self is associated with enhanced reactivity in the insula in individuals with a history of antisocial behavior. Possibly, receiving feedback from others has been experienced as more ambiguous, a question can be addressed by examining the trajectories over time in future research.

#### 2.2.2. Aggression (regulation) in response to social feedback

One way to protect our self-image is by retaliation (Chester et al., 2018), which requires a combination of the input and output steps of the SIP model. Throughout development, people show various compensatory behaviors to maintain positive and coherent self-reviews, like blaming negative feedback on external sources, devaluation of feedback sources (DeWall et al., 2009; Chester et al., 2018), or retaliatory and aggressive behavior (Achterberg et al., 2016; Van de Groep et al., 2021). Especially in the context of reactive aggression, such retaliatory behavior is thought to result from poor cognitive or behavioral control (Bertsch et al., 2020). Limited cognitive control can result in aggression in at least two ways: a lack of response inhibition (i.e., difficulties overriding predominant responses associated with situational cues), and a lack of emotion regulation (i.e., inability to downregulate negative emotions), and aggressive behavior is strongest in individuals who display difficulties in both forms of cognitive control (Bertsch et al., 2020).

Neuroimaging studies have identified several (lateral) fronto-parietal regions that are implicated in cognitive control of aggressive responses, including the dlPFC, dmPFC, vlPFC, OFC, dACC, AI, and preSMA (Grahn et al., 2008; Reidy et al., 2011; Bertsch et al., 2020; Brockett et al., 2020; Crew et al., 2021; van Heukelum et al., 2021). For instance, in typically developing young adults, negative social feedback typically elicits more aggression than neutral and positive feedback, and stronger activity in the dlPFC has been associated with less reactive aggression following negative social feedback (Achterberg et al., 2016; Van de Groep et al., 2021). Moreover, regulating aggressive responses following positive feedback was associated with more activity in the ACC/bilateral Frontal Inferior Triangularis and left middle frontal gyrus (Van de Groep et al., 2021).

To experimentally examine retaliation, the SNAT paradigm allows individuals to blast a loud noise to their peer(s) following feedback. Prior research showed that individuals give the loudest noise blasts following negative feedback, less following neutral feedback and least following positive feedback (Achterberg et al., 2016, 2017, 2018, 2020; Dobbelaar et al., 2021, 2022; Van de Groep et al., 2021; van de Groep et al., 2022a). In our prior research in which we studied how young adults with childhood-limited persistent antisocial behavior, childhood-limited desistant antisocial behavior and typically developing young adults regulated their aggressive behavior following social feedback (van de Groep et al., 2022a), we observed that young adults with a persistent developmental trajectory of antisocial behavior showed similar levels of noise blast aggression as the other two groups following negative feedback. Instead, young adults with a persistent antisocial development did not differentiate in their behavioral responses and showed equally aggressive responses regardless of feedback type, unlike controls and those with a desistant antisocial trajectory. Moreover, after receiving positive feedback, young adults with a persistent antisocial trajectory showed less dlPFC activity during their behavioral response (noise blast delivery), compared to the other two groups. Our findings further revealed that individuals with a desistent antisocial trajectory showed specific behavioral and neural mechanisms that may explain why they manage to successfully desist from antisocial behavior. Recall that in our study, these individuals specifically showed more dlPFC activity during social feedback processing compared to the other groups, which may prepare them to respond adaptively to changing contexts. When examining the subsequent behavior, we found a positive association between aggression regulation following positive feedback and activity in the ACC and dorsal striatum (caudate and putamen) during the behavioral response (noise blast), which was strongest in the desistant antisocial trajectory.

An important question that remains unanswered is which specific aspect of cognitive control (i.e., emotion regulation or response inhibition) is more important in determining the observed differences in the (regulation of) aggressive behavior between developmental groups. Given that the SNAT paradigm does not allow us to dissect the exact cognitive control process that potentially cause these differences, it remains unclear whether the diverging patterns are the result of differences in inhibitory control, emotion regulation, or both. Based on our findings, we hypothesize that young adults with a persistent antisocial history have problems with both emotion regulation (e.g., downregulating their context-independent emotional and neural hypersensitivity) and response inhibition (e.g., failure to inhibit and adapt their prepotent response to react aggressively, regardless of social context). Conversely, individuals with a desistant antisocial trajectory may show similar difficulties in initial emotional responses (i.e., emotional and neural hypersensitivity), but more successful emotion regulation (e.g., attention to changing task demands and reappraisal of salient information) and response inhibition (e.g., inhibiting responses when such behavior is more appropriate, such as following positive or neutral feedback) (Gross and Levenson, 1993). Yet another possibility that should be considered is that the differences in (the regulation of) aggressive behavior are not necessarily caused by deficits in the ability, but rather in the motivation to exert cognitive control (Somerville and Casey, 2010; Buckholtz, 2015; van de Groep et al., 2022a). Future research should further entangle these possibilities, paying close attention to the potential timing and duration of–and interaction between–cognitive control processes (Sheppes and Gross, 2011).

For a long time, researchers have theorized that similar to persistent antisocial behavior, high levels of psychopathic traits likely negatively influence behavioral regulation of aggressive behavior. In line with this idea, a recent meta-analysis indicates that the overall level of psychopathic traits is negatively–albeit modestly–related to response inhibition (Gillespie et al., 2022). Likewise, general psychopathic traits have also been associated with dysregulation of emotions (Garofalo et al., 2018). However, it should be noted that, although some uniformity has been found across psychopathic trait sub-dimensions (Garofalo et al., 2018), other evidence suggests that different subdimensions can be differently associated with emotion regulation and response inhibition (Garofalo et al., 2018; Gillespie et al., 2022). For instance, some studies highlight that Impulsive-Irresponsible traits in particular may be associated with emotion dysregulation, while no evidence was found for an association between Callous-Unemotional traits and emotion regulation (Long et al., 2014; Preston and Anestis, 2020). At the same time, other researchers hypothesize that in some social situations, Callous-Unemotional traits may be associated with dysfunctional emotion regulation, which is likely not result of difficulties to control strong emotions (like Impulsive-Irresponsible traits), but rather of limited awareness and acceptance of their emotions (Garofalo et al., 2021). Hence, future studies should disentangle to what extent difficulties in emotion regulation and response inhibition are driven by general psychopathic traits, or (interactions between) specific sub-dimensions (not only focusing on Impulsive-Irresponsible and Callous-Unemotional, but also Grandiose-Manipulative traits) (Lilienfeld, 2018), to further unravel the etiology and maintenance of antisocial behavior (Gillespie et al., 2022). In this regard, it is important to consider different components of response inhibition and emotion regulation, and to combine self-report measures with experimental fMRI tasks to uncover the neural underpinnings of cognitive control that are specific for psychopathic traits and persistent antisocial behavior (Korponay and Koenigs, 2021).

#### 2.2.3. Updating beliefs about the self and one’s goals

Although people may protect their self-beliefs after receiving social feedback, they can also decide to use the feedback to update their self-concept and goal representations (Elder et al., 2021; Crone et al., 2022), which increases their self-efficacy and self-control in future situations (Jiang et al., 2022). From a SIP perspective, goals are mental representations that influence all social information processing steps (Crick and Dodge, 1994), and orientate people toward producing or wanting (to avoid) particular outcomes that have not been attained yet (Crick and Dodge, 1994; Moskowitz, 2012). These social-cognitive structures thus specify the desired outcomes (i.e., end states) one wants to attain, and the means (i.e., which actions result in the desired outcome) and motivation (i.e., anticipatory desire) to attain this end state (Moskowitz, 2012). Striving toward a goal involves the detection of discrepancies between the current state and the desired end state or standard (note that the self-concept is an important standard) (Carver and Scheier, 1981). As we have seen before, the source of these discrepancies can be an external agent providing social feedback. Importantly, if a discrepancy is detected, and the provided social feedback is judged as relevant, the mental representations of the self (i.e., self-concept) and one’s goals can be updated. Likewise, actually attaining self-relevant goals also reinforces both the positivity and clarity of how we see ourselves (Lavallee and Campbell, 1995; Ayduk et al., 2009). Examples of self-relevant goals that influence updating beliefs about the self include the goal to maintain a positive self-image (i.e., self-enhancement), to maintain a consistent and coherent view of the self (i.e., self-verification) and to achieve an accurate, realistic image of the self (i.e., self-assessment) (Neiss et al., 2006).

Typically developing young adults generally update their self-beliefs in line with the motives to maintain a positive, consistent and coherent self-image. Accordingly, they show changes in self-concept in line with positive, but not negative feedback (Korn et al., 2012; Rodman et al., 2017; Elder et al., 2021). Research suggests that the (v)mPFC may facilitate positive biasing of self-concept updating (Will et al., 2017; Yoon et al., 2018; Elder et al., 2021, 2022; Zamani et al., 2022; Zhang et al., 2022). More specifically, the vmPFC may underlie detection whether social information is congruent with existing information (i.e., one’s self-concept, which is usually positive; Elder et al., 2022; based on both immediate feedback and prior social experiences (Yoon et al., 2018). This biasing toward positivity seems to depend on the self-relevance (or integrality and centrality) of the positive feedback for the self-concept (Elder et al., 2021, 2022). In other words, only for relevant and central traits that are congruent with existing information, the self-concept will be “updated” (note that updating here means strengthening of the existing knowledge structure, rather than valence-based changes in self-beliefs).

Conversely, young adults with high levels of psychopathic traits may be biased toward the motive to achieve a realistic image of the self, or have difficulties updating beliefs about the self. Recall that in our self-appraisal task, individuals with higher level of psychopathic traits, but not necessarily with persistent antisocial trajectories (i.e., showing ASPD) show more negative prosocial (but not physical) self-evaluations. This could reflect a realistic view of their social selves, informed by prior social experiences. However, if this was the case, one might have expected to observe the same findings for persistent antisocial behavior and high levels of psychopathic traits. Alternatively, young adults with high levels of psychopathic traits may show difficulties with integration (updating and accumulation) of social feedback to form a positively biased social self-concept. Indeed, individuals with psychopathy may be worse at integrating and updating accumulated (social feedback) information (Hamilton et al., 2015), which affects the positivity, coherence and stability of their self-concept (Doerfler et al., 2021; van de Groep et al., 2022b). A lower self-concept clarity, in turn, further limits individuals propensity to incorporate new information into their self-concept after new social experiences (i.e., self-expand by adding information about new identities, knowledge and social roles) to avoid the risk that conflicting information reduces self-concept clarity even further (Emery et al., 2015). Information integration and self-expansion in psychopathy is likely further hampered by an early attentional bottleneck that facilitates processing of goal-relevant, but not other salient situational and relational cues, that are ultimately important for behavioral adaptation (Baskin-Sommers and Newman, 2013; Baskin-Sommers and Brazil, 2022). Future research should examine this possibility in more detail, to disentangle whether difficulties in motivation or ability to update information into the self-concept are specific psychopathy (both the overall construct and respective sub-dimensions) (Baskin-Sommers and Newman, 2013).

### 2.3. Learning how behavior may benefit self and others

Although people sometimes detect goal-related discrepancies through social, external feedback, it most often results from internal monitoring of whether actions result in the desired end states (Moskowitz, 2012). Adaptive social responses in rapidly changing social environments require young adults to learn how their actions and the associated outcomes are valuable or aversive for themselves, but also for other social agents at the same time (Christopoulos and King-Casas, 2015; Cutler et al., 2021). Individuals learn action-outcome associations over time, through repeated iterations, known as reinforcement learning (Sutton and Barto, 2018). From a SIP perspective, learning such action-outcome associations forms the basis of potential (dominant) behavioral responses that will be selected from memory in the future, and helps young adults to evaluate whether they should select and implement a response, based on the anticipated consequences of their behavior, which both support the goal they wish to attain (i.e., SIP steps 3–6).

Adaptive social behavior has previously been examined using probabilistic, or reinforcement learning tasks. While performing these tasks, individuals are required to make a series of choices, where each option probabilistically results in positive, negative or neutral outcomes (Nussenbaum and Hartley, 2019). The probability of these outcomes can remain stable throughout the task, or change at specific moments (Nussenbaum and Hartley, 2019). Over the series of choices, individuals thus learn what the best option is, and when they should change their behavior (e.g., when an option is no longer rewarding). Typically developing young adults learn to repeat actions that result in positive, rewarding and valuable outcomes from themselves, while negative outcomes like losses are often avoided (Carvalheiro et al., 2021). During the transition from adolescence to young adulthood, reinforcement learning becomes more optimal (Nussenbaum and Hartley, 2019), with individuals showing improvements in weighing and interpretation of both rewards and losses, combined with an increased ability to integrate appropriate information into their reinforcement history (Javadi et al., 2014). Accordingly, young adults improve their ability to rely on more goal-directed, model-based learning (i.e., not solely based on action-outcome associations, as model-free learning) (Cutler et al., 2022).

One question that arises is how individuals learn to develop a long-term strategy when learning in a probabilistic environment. Compared to children and adolescents, young adults are less likely to persist in behaviors that are no longer rewarding (i.e., they show proficient reversal learning skills), like showing aggression in a changed social context (Decker et al., 2016; Eckstein et al., 2022). Moreover, young adults increasingly consider how their actions influence not only their future self [e.g., prioritizing long-term over short-term goals (Mischel et al., 1989)], but also others (Monahan et al., 2013; Cutler et al., 2021), and increasingly show social actions that benefit others (i.e., prosocial behavior), which ultimately also benefit themselves, by enhancing one’s own mental and physical wellbeing (Cutler et al., 2021). Still, young adults are generally better at learning for themselves than for others (Lockwood, 2016; Cutler et al., 2021; Martins et al., 2022).

A further way to examine the mechanistic underpinnings of reinforcement learning is by examining neural responses to learning signals. Prior studies using probabilistic learning tasks have shown repeatedly that the striatum is involved in tracking social reinforcement learning signals for self and others while individuals receive outcomes (Lockwood, 2016; Westhoff et al., 2021), particularly when receiving rewards (Dugré et al., 2018; Oldham et al., 2018), while losses are associated more closely with the dorsal striatum (Dugré et al., 2018; Murray et al., 2022). Another area that has been implicated in reinforcement learning while receiving outcomes for self and others is the PFC (Khamassi et al., 2013; Javadi et al., 2014; Lockwood and Wittmann, 2018; Westhoff et al., 2021), both while receiving rewards (Lockwood and Wittmann, 2018) and losses (Dugré et al., 2018). Previous studies further suggest that rostral and subgenual regions of the mPFC might be specialized in learning for others in social contexts (Christopoulos and King-Casas, 2015; Lockwood, 2016). Taken together, typically developing young adults become increasingly proficient in learning about and monitoring actions of the self, while adjusting to the demands of the specific social context.

Considering reinforcement learning and internal monitoring processes may provide us with important information on how individuals with a history of antisocial behavior learn in and adapt to a changing social world. Recently, researchers have argued that transdiagnostic reinforcement learning difficulties may underly various antisocial tendencies and behaviors, including persistent antisocial behavior (e.g., ASPD) and psychopathy (Pauli and Lockwood, 2022). While there is some evidence in support of this notion, the specificity of these effects likely depends on the valence of the outcomes (Murray et al., 2018). For instance, persistent and more impulsive antisocial behavior may arise from deficits in responding to and learning from various forms of incentives like rewards and losses (Murray et al., 2018). In line with this idea, impulsive antisocial tendencies are associated with neural hyperresponsivity when processing reward-related cues in the ventral striatum and PFC/ACC (Murray et al., 2018), particularly during anticipation of rewards, but also during reward receipt. Likewise, individuals with impulsive antisocial tendencies show stronger neural activity during processing of punishments/losses (Murray et al., 2018). Together, these findings fit with the idea that persistent and impulsive antisocial behavior is associated with increased reactivity to salient feedback information (Baskin-Sommers and Newman, 2013; Murray et al., 2018; van de Groep et al., 2022a). Note that regarding the actual learning from outcomes, as of yet, few studies in high-risk or forensic samples go beyond identifying responses to incentives and thus, the neural basis of reinforcement learning remains largely unclear (Pauli and Lockwood, 2022).

In the context of probabilistic learning, a focus on individual differences has been promising to discern heterogeneity in antisocial tendencies. That is, more affective psychopathic traits (e.g., Callous-Unemotional traits) seem predominantly associated with deficits in learning from punishments and losses (von Borries et al., 2010; Murray et al., 2018), rather than rewards. The super-ordinate construct of psychopathy has also been associated with slower learning (Moul et al., 2021) and a failure to learn from negative consequences (von Borries et al., 2010). Reinforcement learning studies in a subclinical population suggest that individuals with higher levels of psychopathic traits fail to update action-outcome values after avoiding a negative result (Oba et al., 2019), once again signaling difficulties with the updating and integration of feedback information. Likewise, psychopathy has been associated with difficulties adjusting behavior when behavior no longer results in a reward (e.g., reversal learning), but also with an increased propensity to change behavior following a rewarding outcome (Blair, 2013). During reversal learning, psychopathy has been associated with increased neural activity in the posterior cingulate and AI (Gregory et al., 2015). However, little is known about the neural basis of reinforcement learning in young adults with higher levels of psychopathic traits, given that computational reinforcement learning models have only focused on adolescence so far (White et al., 2013; Brazil et al., 2017).

A salient developmental feature of early adulthood is the ability to balance self-relevant goals with an increased consideration of others, to establish and maintain mature relationships (Nelson et al., 2008). However, existing studies on reinforcement learning and psychopathy or antisocial behavior, especially those involving neuroimaging methods, have largely focused and learning for the self, rather than for self and other (simultaneously) (Christopoulos and King-Casas, 2015; Cutler et al., 2021). In previous studies in healthy (young) adults that focused on learning in social contexts, more antisocial behavior and higher psychopathic traits, were both associated with reduced sensitivity for the outcomes of others (Cutler et al., 2021; O’Connell et al., 2021). However, it remains unclear whether and how these findings translate to more high-risk and forensic samples. Moreover, the finding that in healthy young adults, reinforcement learning relies on partly overlapping, but partly distinct neural substrates raises the question how motivation influences reinforcement learning, and how different competing self-relevant goals (i.e., maximizing rewards for self and others) may be implemented and updated (Christopoulos and King-Casas, 2015; Lockwood, 2016) in young adults with antisocial and psychopathic tendencies.

## 3. Working model and future directions

**FIGURE 2:**
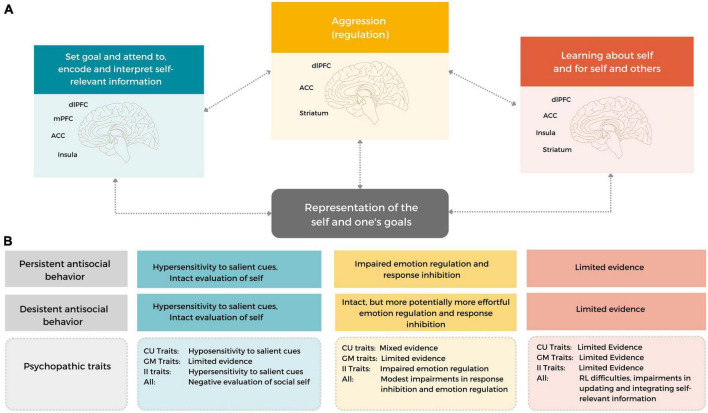
A neurocognitive working model of early onset persistent and desistant early adulthood antisocial behavior. The working model includes several neurocognitive functions that can be used to understand aggressive, antisocial behavior in current situations (i.e., state-related processes) and throughout the course of development (by incorporating how social information is updated and integrated to guide future behavior and neural processing) and in changing social contexts. Importantly, the model incorporates developmentally salient features of early adulthood, related to understanding and monitoring one’s self-traits and goals. **(A)** A heuristic depiction of the described functions and their development [i.e., (1) goal setting and attending to, encoding and interpreting self-relevant information (SIP steps 1–3; input-related steps), (2) monitoring, updating and integration information] (SIP steps 3–6, output related steps) and (3) Aggression (regulation) (SIP step 5–6). These functions rely on (ongoing development in) frontal and subcortical limbic brain areas, including the dlPFC, (v)mPFC, ACC, Insula, Amygdala and Striatum. **(B)** Individuals with an early onset, persistent antisocial development (likely) show some similarities, but also marked differences in neurocognitive functioning to young adults with a desistant trajectory. In addition, both the overall construct of psychopathy and separate dimensions of psychopathic traits may help to differentiate between heterogenous antisocial expression in different social contexts and throughout development.

In this review, we brought together the existing empirical literature to examine which behavioral and neurobiological social information processes may underlie differences in the development and maintenance of, and desistance from aggressive behavior in early adulthood. To this end, we focused on developmentally salient features specific to early adulthood: how young adults evaluate, act upon and monitor and learn about their goals and self traits. Based on the reviewed literature, we formulate a neurocognitive working model for early onset persistent and desistant early adulthood antisocial behavior (see Figure 2), that can be used as a framework for future studies, and highlight important considerations for future research.

Persistent antisocial behavior in early adulthood is characterized by impairments in self-relevant and goal-related (feedback) information processing, aggression regulation, and potentially in the monitoring, updating and integration of self-relevant information. These impairments are associated with dysfunction in several frontolimbic brain regions, including the (v)mPFC, dlPFC, ACC, Insula, Amygdala and Striatum (see Figure 2), although other brain areas might be involved. The neurocognitive difficulties in persistent antisocial behavior seem to be characterized by a limited capacity to differentiate between differently valenced cues and to adapt their behavior to specific and changing social contexts. Although desistant antisocial development is likewise associated with neural hypersensitivity to salient, self-relevant social feedback information, it is likely related to intact, but perhaps more effortful, aggression regulation and monitoring, updating and integration of information – which allows for successful behavioral adaptation in different social contexts.

Although some neurocognitive functions are likely impaired in both persistent antisocial behavior and psychopathy (e.g., reinforcement learning), others seem specific to the overall construct of psychopathic traits (e.g., self-evaluation; updating and integrating of social information). Hence, individual differences in (subdimensions of) psychopathic traits may amplify or attenuate, or be differentially associated with difficulties in neurocognitive functions associated with self-relevant and goal-related social information processing. In this respect, difficulties implicated in psychopathy may specific or more pronounced for negatively valenced cues [self-evaluation, updating, monitoring and integration of (pro)social self-relevant information]. Taken together, these observations highlight that studying both the general construct of psychopathy, as well as its respective sub-dimensions may help to differentiate between heterogenous antisocial expressions in different social contexts and throughout development.

It is important to note that the current review and working model point toward four open questions about self-relevant and goal-related social information processing in early adulthood antisocial behavior. First, research on most of the hypothesized neurocognitive functional impairments (and in particular on reinforcement learning) and psychopathic trait subdimensions (particularly Grandiose-Manipulative traits) in early adulthood is still in its infancy, and warrants further consideration and replication–particularly in high-risk groups. Notably, most results on persistent and desistent development presented here were based on the same (single) sample (i.e., the RESIST Study, see van de Groep et al., 2022a,b), highlighting the need to conduct research in additional developmental samples. Second, with regard to goal-directed behavior, research on SIP has mainly focused on impaired neurocognitive functions associated with the processing of outcomes and the means to attain these specific outcomes. As of yet, much less is known about the specific motivation to reach one’s goal(s) in psychopathy and persistent antisocial behavior. Thus, for all hypothesized impairments in neurocognitive functions described in the working model, it should be clarified whether they result from an impaired ability or motivation, or both. Third, we mainly focused on one specific individual difference that can influence the expression and development of antisocial behavior (psychopathic traits). However, we acknowledge that there are many other important and relevant individual differences that could be incorporated more comprehensively into the working model–including, but not limited to gender (Eme, 2020), trauma/maltreatment [for meta-analyses, see Braga et al., 2017; de Ruiter et al., 2022 and (comorbid) clinical diagnoses]. Fourth, like individual differences, environmental factors have been shown to play an important role in the development and persistence of antisocial behavior (e.g., Moffitt, 2018), highlighting the need to further investigate the interaction between the environment and social-cognitive processes.

Based on the current literature review, we highlight four important and related considerations for future research on antisocial development in early adulthood more broadly. First, it is important gain a more comprehensive understanding of differences (in stability) between–and changes within–antisocial developmental pathways. To this end, future research should go beyond cross-sectional research in early adulthood, and conduct longitudinal studies across development to help identify the onset, rate and consistency of the developmental processes of interest and corresponding neural underpinnings. Longitudinal research may also reveal insights into the causality and temporal order of developmental and life events, and thus provide starting points to understand potential mechanisms of change–which is essential to develop suitable and personalized interventions. Such research may also help to identify whether differences in brain development are causally related to antisocial behavior – and may be either the cause or the consequence of antisocial behavior–or both. Importantly, pointers for change may also be provided by focusing on more immediate, short-term and dynamic adaptations in social contexts (Flechsenhar et al., 2022). For instance, focusing on time-related changes within tasks, and trial by trial changes may further illuminate how the brain computes processes underlying antisocial behavior (Bertsch et al., 2020; Pauli and Lockwood, 2022), and facilitates the development of both social competence and personal goal attainment (Flechsenhar et al., 2022). Taken together, longitudinal studies and trial-based analyses may shed new light on the exact timing of SIP difficulties, and opportunities for both immediate (short-term) and developmental (long-term) adaptations in social contexts (Flechsenhar et al., 2022).

Second, the literature we reviewed above clearly stresses the importance of considering the complex interaction between characteristics of the social context, the aggressive or antisocial response and individual characteristics to understand the neurodevelopment of antisocial behavior. Different aspects of the social context (e.g., the specific trigger of antisocial behavior - social rejection, frustration or threat) and how many people are involved (Bertsch et al., 2020; Rappaport and Barch, 2020), from which specific target group [e.g., friends, (delinquent) peers, parents] determine how and why people act in a specific manner (e.g., showing reactive aggression in response to social rejection, based on the affordances of the situation), in interaction with individual characteristics (e.g., achieving self-relevant goals in line with one’s self-concept, goal representations and personality). Moreover, the adaptivity of antisocial behaviors and tendencies likely changes over time and as contexts change [e.g., when growing up in a hostile environment, these tendencies might be adaptive (at least in the short term), but cease to do so when a variety of different contexts with changing demands and affordances are encountered in adolescence and young adulthood]. However, as of yet, many studies, including the ones reviewed in the current article, focus on establishing 1:1 associations between factors, such as neurobiological factors with behaviors (Beauchaine and Hinshaw, 2020). Thus, an important avenue for future research is to incorporate different social-cognitive, neurobiological and environmental measures into one (longitudinal) approach (Brazil et al., 2018; van der Wal et al., 2021; Carbonneau and Tremblay, 2022; Jansen, 2022), to do justice to the complexity of these factors and their interplay. Such integrative approaches require a large amount of data, and thus, studies with large sample sizes. Given that antisocial populations are difficult to recruit and retain, especially in neuroimaging research, these approaches will likely involve the merging of datasets within consortia (Brazil et al., 2018; see for example the ENIGMA project, Thompson et al., 2017). However, it is important to note that although this approach may help to understand the heterogeneity in the display and development of antisocial behavior, much about the (development of) functional mechanisms of interest underlying antisocial development is still unclear. Thus, such approaches should be complimented with targeted (fMRI) studies that aim to identify or clarify (changes in) important functional, computational, behavioral mechanisms - or situational and personal characteristics. Ultimately, a combination of these approaches, which relies on their combined strengths, is most likely to advance our understanding of (the development of) antisocial behavior (Brazil et al., 2018).

Third, it is important to consider the arguably complex role of psychopathic personality traits in the neurodevelopment of (persistent) antisocial behavior in more detail. The studies reviewed above highlight that there might be both overlapping and distinct features of persistent antisocial behavior and psychopathic trait dimensions, that can be used to differentiate between diverse impaired functional and neurobiological social information processing mechanisms (Lilienfeld, 2018; Pauli and Lockwood, 2022), and help further explain heterogenous pathways in antisocial development. Between the specific psychopathic dimensions, the reviewed literature also reveals evidence for both overlapping and distinct functional and neurobiological mechanisms (Garofalo et al., 2018; Gillespie et al., 2022; van de Groep et al., 2022b). To improve our understanding in the differences and overlap between persistent antisocial development and psychopathic traits, it is important to also consider how changeable and stable both psychopathic traits and antisocial behavior are–throughout development and across different social contexts. Both persistent antisocial behavior and psychopathic traits are assumed to involve a certain stability, and traditionally, psychopathic traits have been assumed to be relatively insensitive to change throughout development (Nentjes et al., 2022). Although for most individuals, psychopathic traits remain quite stable during the transition from adolescence to young adulthood (Lee and Kim, 2020), there is also evidence that this is not always the case, and not always the same for all psychopathic trait subdimensions [especially Grandiose-Manipulative traits seem susceptible to change (Lee and Kim, 2020)], and that impairments are not always present across different social contexts (Nentjes et al., 2022). A related question is how central the reviewed difficulties are to psychopathic traits and persistent antisocial development (Garofalo et al., 2018; Gillespie et al., 2022). Thus, future research should examine this centrality, and consider how environmental influences impact the nature and stability of antisocial and (global and dimensional) psychopathic traits throughout development (Blair, 2013), across different social contexts.

Fourth, it is important to gain a better understanding of sex/gender differences in the development of antisocial behavior, especially in early adulthood (Cauffman et al., 2015; Lanctôt, 2015; Wierenga et al., 2023). Antisocial behavior in males is typically more prevalent and severe than in females (Cauffman et al., 2015; Lanctôt, 2015) and males are 10–14 times more likely to develop life-course persistent antisocial behavior than females (Eme, 2020). Yet, some research in children and adolescents suggests that the extent to which such a sex/gender-gap arises depends on developmental stage and the type of antisocial behavior being assessed (Lanctôt, 2015). Moreover, female antisocial behavior may be associated with different risk factors (e.g., exposure to violence, mental health problems, Cauffman et al., 2015), as well as different shapes of developmental trajectories (Fontaine et al., 2009; Lanctôt, 2015). As such, future studies should explore the extent to which a sex/gender gap is present in early adulthood antisocial development. Important directions include (1) differentiating between different sex/gender-sensitive constructs, such as risk factors and specific types of antisocial behavior, and (2) using sample sizes with a large number of females and/or individuals who identify as female or have other non-men gender identities (Fontaine et al., 2009; Lanctôt, 2015; Freitag et al., 2018; Wierenga et al., 2023).

## 4. Conclusion

To conclude, our review showed that it is important to consider developmental aspects of early adulthood to understand functional and neurobiological mechanisms that underlie different developmental trajectories of antisocial behavior. This review offers a comprehensive perspective that includes self- and other related processing by considering how young adults evaluate, act upon, monitor and learn about their goals and self traits. As such, this perspective provides valuable starting points to understand how and why some individuals with an early onset of antisocial behavior manage to adapt to changing social environments and balance between situational characteristics and self-relevant goals and motivations, while others fail to do so and persist in antisocial behavior. Importantly, our review also shows that considering functional neuroimaging alongside behavior reveals new insights that help to overcome difficulties (e.g., biases) associated with behavioral (self-report) measures. Finally, considering individual differences such as psychopathic traits, and specific emotional characteristics (e.g., valence of self-traits and goal-related feedback) may further illuminate functional and neural mechanisms underlying heterogenous developmental pathways. Additional research should examine changes and stability in antisocial and psychopathic tendencies throughout development and between different social contexts to further clarify whether functional neurocognitive deficits related to the development of antisocial behavior are general or context- and valence specific – and central to antisocial development in young adulthood. Ultimately, such research will provide important advances required to understand and overcome persistent antisocial behavior, and provide starting points for the development of timing-appropriate and personalized interventions.

## Author contributions

IG wrote the manuscript with the help of MB, AP, EC, and LJ. All authors contributed to the article (writing, editing, and supervision) and have read the submitted manuscript.
